# Simultaneous Segmentation of Leukocyte and Erythrocyte in Microscopic Images Using a Marker-Controlled Watershed Algorithm

**DOI:** 10.1155/2018/7235795

**Published:** 2018-02-22

**Authors:** Huisi Miao, Changyan Xiao

**Affiliations:** ^1^College of Electrical and Information Engineering, Hunan University, Changsha, China; ^2^Kunshan Hunan University Robot Technology Co., Ltd., Kunshan, China

## Abstract

The density or quantity of leukocytes and erythrocytes in a unit volume of blood, which can be automatically measured through a computer-based microscopic image analysis system, is frequently considered an indicator of diseases. The segmentation of blood cells, as a basis of quantitative statistics, plays an important role in the system. However, many conventional methods must firstly distinguish blood cells into two types (i.e., leukocyte and erythrocyte) and segment them in independent procedures. In this paper, we present a marker-controlled watershed algorithm for simultaneously extracting the two types of blood cells to simplify operations and reduce computing time. The method consists of two steps, that is, cell nucleus segmentation and blood cell segmentation. An image enhancement technique is used to obtain the leukocyte marker. Two marker-controlled watershed algorithms are based on distance transformation and edge gradient information to acquire blood cell contour. The segmented leukocytes and erythrocytes are obtained simultaneously by classification. Experimental results demonstrate that the proposed method is fast, robust, and efficient.

## 1. Introduction

The number and type of blood cells must be counted in routine peripheral blood and bone marrow cell diseases [1]. Traditional manual methods require haematologists to carefully observe the morphological characteristics of leukocytes and erythrocytes under a microscope. This process heavily depends on their skills and experiences, and the morphological characteristics may be misjudged and missed. Computer-aided systems based on digital image processing can reduce the erroneous judgment of human factors, improve inspection efficiency, and assist examiners in making decisions [2]. The steps in these expert systems are separating target cells from cell clusters or image background, extracting the feature vector of the cells, and classifying unidentified cells by a trained classifier. Segmentation results directly affect the accuracy of cell classification and counting and play an important role in the expert systems. The background of blood smear image is complicated, and cells significantly overlap with one another. Consequently, complete cell extraction is difficult to realise and has become a new topic in medical image processing.

Several works on microscopic segmentation of blood smear images are available in the literature. Most of the works have focused on leukocyte (also referred to as white blood cell, WBC) segmentation, whereas limited research is available on erythrocyte (also called red blood cell, RBC) segmentation algorithms. This inequality is attributed to two main reasons. The first reason is that WBCs which are called immune cells and can help the body fight infection and external substances are more important in disease diagnosis than RBCs. The second reason is the high density of RBCs in blood, the complex distribution of RBCs, the variation in RBCs in blood smear images, and the difficulty in using traditional segmentation methods.

Several approaches have been developed for WBC segmentation. In principle, the methods can be categorised into three classes: threshold-, learning-, and deformable mode-based methods. Zhang et al. [3] proposed a method for segmenting WBCs on the basis of a colour enhancement technique and Otsu's threshold. Huang et al. [4] developed circle detection to obtain complete cells. Cuevas et al. [5] used a combination of* k*-means algorithm and colour space transformation to segment the WBCs in leukocytes and blast cells. Sadeghian et al. [6] presented a WBC segmentation framework that segments the cytoplasm by using a gradient vector flow (GVF) snake.

Similarly, studies that have worked on RBC segmentation methods have been documented. For example, Ma et al. [7] presented a new method based on the quality of binary images preprocessed using a Pulse Coupled Neural Network (PCNN) to infer RBC contours. Rashid et al. [8] used moving* k*-means clustering algorithm to segment an unsupervised colour image of thalassemia disease. Wei and Cao [9] segmented overlapping cells by combining region-growing algorithm and colour space transformation.

The above-mentioned algorithms are aimed at obtaining only WBCs or RBCs in blood smear images. No cell segmentation method that can simultaneously acquire WBCs and RBCs in blood smear can be used for analysing the two types of cells in experiments. A segmentation method for blood cell images is presented in this paper. This method realises the simultaneous segmentation of WBCs and RBCs in the blood smear images and can simplify operations and save time.

## 2. Background and Challenges

The blood smear images from a microscope provide important information for diagnosing and predicting diseases in the haematological analysis. Blood samples are prepared and sent to a blood cell counter for calculating each cell type. If haematologists find an unusual number of cells in any type, then they will investigate further by exploring the microscopic blood smear, recounting the number of cells and checking their morphology in detail. Any blood cells with irregular shapes or characteristics may trigger severe diseases. The visual inspection by haematologists is tedious and time-consuming. Therefore, an automating process is highly desirable to accelerate the process. Three types of blood components, that is, RBCs, WBCs, and platelets (PLTs), are present in a blood smear. The RBCs transport oxygen from lungs to all living tissues in the body and carry carbon dioxide. The RBCs are normally found in up to 40%–50% of the total blood volume. The diameter of RBCs is 6–8 *μ*m. The WBCs play an important role in the immune system of the body by defending it against infectious diseases and foreign materials. Therefore, an analysis of the WBC and RBC characteristics is essential.

**Figure 1:**
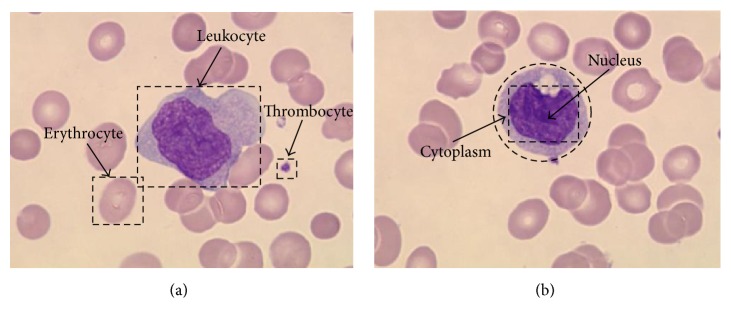
Sample blood smear images.

A successful solution of cell segmentation requires incorporating domain-specific characteristics of images into the segmentation algorithm, similar to many segmentation problems.  Figure 1 demonstrates two sample images containing different cell types, each of which has its own characteristics. WBCs come from the nucleus and cytoplasm. Mature RBCs are nonnucleated, biconcave disc-shaped cells, and PLTs exhibit a colour that is similar to the WBC nuclei and an irregular shape. The purple-like colour of WBCs typically makes them easy to differentiate from RBCs and the background. However, false positives might be produced when the RBCs are abutting these cells because their boundary regions may show similar colouration.

In the blood smear images, WBCs and RBCs are adhered to each other, the cells overlap, and the image background is complex. These features are challenging for image segmentation algorithms. The traditional image segmentation methods are invalid to these images and cannot get the satisfactory results. In addition, these methods often need to first distinguish blood cells from two different types, namely, WBCs and RBCs, and, respectively, segment them in independent procedures. It makes the process of image processing more tedious and time-consuming. From the above viewpoint, we try to make the process of blood cell image segmentation more simple and effective.

## 3. The Proposed Segmentation Algorithm

In the blood cell images, stained cell nucleus, WBC cytoplasm, and RBCs present visual differences. We divide the blood cell segmentation algorithm into two major steps. In the WBC nucleus segmentation step, a cell nucleus enhancer is proposed to segment the region we are interested in by enhancing the region of the leukocyte nucleus and suppressing the other region of the blood smear image.

**Figure 2:**
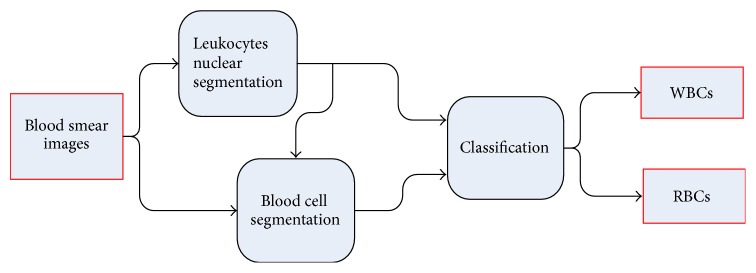
Flowchart of the proposed method.

We must separate the WBC cytoplasm region and RBCs from the background of the image in the blood cell segmentation step. The cell extraction process can be considered the image foreground and background of the two classification problems because the grey values of the background and other regions in the image are large and easy to distinguish. This step usually consists of two procedures. One procedure is separating cells from the background or the interfering part to generate the binarised image of the cells, where the target cell region is 1. The other procedure is separating cells, including the separation of adherent cells. This step is typically difficult.  Figure 2 depicts the proposed framework on the segmentation scheme.

### 3.1. Leukocytes Nucleus Segmentation

**Figure 3:**
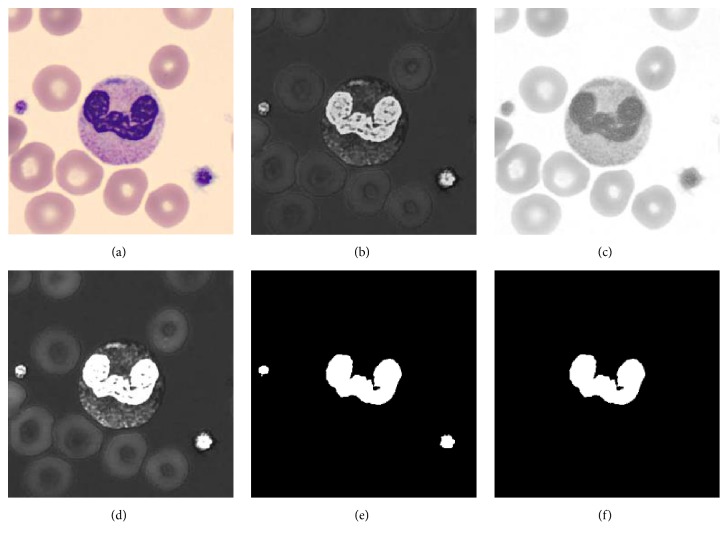
Flowchart of leukocytes nucleus segmentation: (a) original image, (b) *S*-channels image, (c) *I*-channels image, (d) IMG_1_ [the result after formula (1)], (e) the binary image of nucleus, and (f) the binary image after postprocessing.

The WBC nucleus region is equivalent to the WBC marker; that is, WBC nucleus and WBC marker exhibit a one-to-one correspondence. The nuclei in the WBCs are markedly different from the other parts of the image given their physical and chemical properties after the blood cells are stained. In Figure 3(a), test images with 360 × 360 sizes are captured by a charge-coupled device (CCD) camera with 100 times objective lens. Blood smear image-staining density can be described by the saturation colour component (Figure 3(b)), where a bright image indicates a high colour saturation value. The stained nuclei have a high value on the *S*-channel and are bright in the image. By contrast, the cytoplasm region, RBCs, and image background have a low grey value and are dark in the image.

The grey distribution of the nuclei in the *I*-channel (Figure 3(c)) is frequently opposite to that in the *S*-channel. Thus, image enhancement techniques are used to extend the dynamic range of the histogram and distinguish the nuclei from other parts of the original image. The two channels can be combined using the following formula:

(1) $$\begin{array}{c} IMG_{1}=\frac{S}{I}, \end{array}$$

where IMG_1_, *S*, and *I* represent the enhanced image, *S*-channels, and *I*-channels of the original image in colour space, respectively. Processing of pixels point by *S* and *I* images in the formula, as shown in Figure 3(d), is easier to extract in nucleus region of IMG_1_. Because the *S*-channel information and the *I*-channel information are independent of the colour, the method is more robust and will not be affected by other staining schemes. Note that the unit unified step of formula (1) is omitted.

By analysing the IMG_1_ grey histogram, the cytoplasmic region, the image background, and the RBCs and the nucleus region formed two distinct peaks on the histogram. Otsu's threshold segmentation method can be used to obtain the nucleus binary map, as shown in Figure 3(e). Notice that the platelet and WBC nucleus have colour similarity to cause platelet to be misclassified into the WBC nucleus. This paper does not discuss the platelet, which can be removed by postprocessing based on regional thresholds. The postprocessing result is shown in Figure 3(f).

### 3.2. Blood Cells Segmentation

The blood cells segmentation method delineates cell boundaries by using a priori shape and edge information obtained from the distance and gradient maps and combining them in a marker-controlled watershed algorithm two times. To this end, it first defines a set of markers as foreground maker on the distance map, where each marker corresponds to an estimated location of a single cell, which is defined as a combination of the intensity and distance maps, in the flooding process of the watershed. At the end, it takes the ridge line obtained from the previous watershed transformation as a background marker. These markers correspond to the background of the image, combine the foreground markers with the background markers to reconstruct the edge gradient topographic map, and get the smooth cell boundary through the second watershed.

As shown in Figure 5, the proposed cells segmentation could be divided into four main steps: binary image extraction, foreground mark extraction, background mark extraction, and gradient topographic map reconstruction. The details are explained in the following subsections.

#### 3.2.1. Binary Image Extraction

Cells in blood smear images overlap with one another. Consequently, correct separation results are difficult to obtain when the original image is directly used for subsequent processing. A gradient image reflects the grey level change information of the pixel. The edge of blood cells in the gradient image is presented as a significant highlight value different from the image background; this image background can be considered the topographic map in the watershed operation and as a priori knowledge of image binarisation.

**Figure 4:**
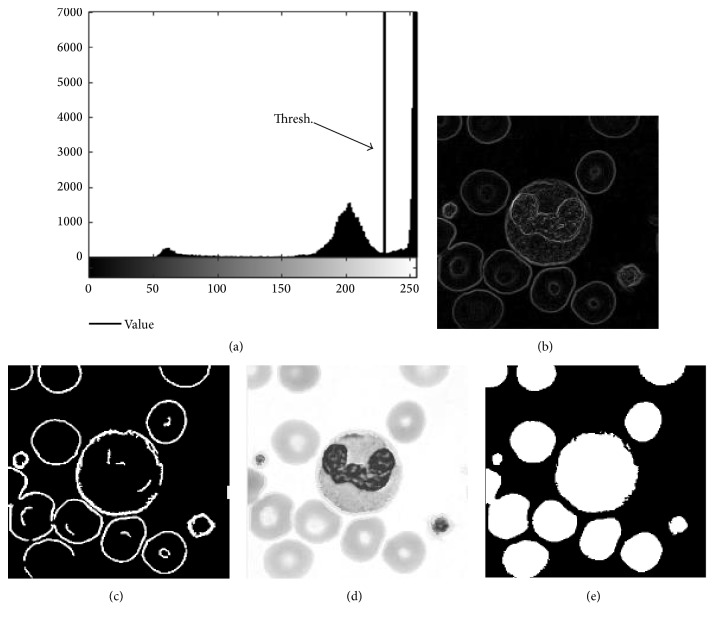
Flowchart of cell binarisation: (a) grey histogram of *I*-channel in cell image, (b) edge gradient image of cells, (c) image of cell contour through a gradient threshold, (d) *I*-channel cell image modified by cell contour, and (e) binary image of cell.

**Figure 5:**
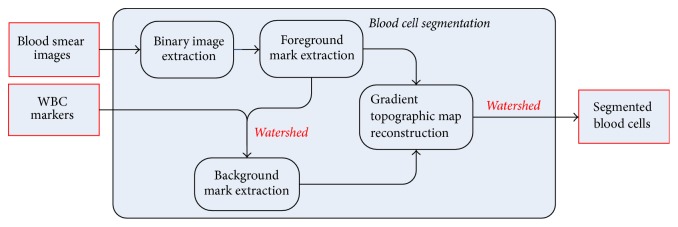
Flowchart of the blood cell segmentation.

According to the above discussion, we can use Sobel edge operator [10] in obtaining the gradient image of the cell (Figure 4(b)) and ∇*I* to represent it. The cell edge in ∇*I* is easy to distinguish in grey scale images and can be obtained by the threshold segmentation operation, as displayed in Figure 4(c). In this step, we obtain the key material of the binarisation operation, that is, the cell edge, which can be separated through the cell edge when the cells overlap. Burrs do not affect subsequent image manipulation, despite many burrs in the cell edge.

The left histogram in Figure 4 shows the *I*-channel grey value distribution of the blood cell image. We can select the extremes of the first valley as a threshold to replace the cell edge by analysing the grey histogram. Thus, we combine the cell edge and image background into a new image (Figure 4(d)), and the binary mask ∇*B* is obtained through threshold segmentation and filling operations (Figure 4(e)).

Gradient images ∇*I* and ∇*B* are obtained through a previous work. These images can be used to extract foreground and background markers on the basis of marker watershed transformations.

#### 3.2.2. Foreground Mark Extraction

Marker-controlled watershed [11] is proposed to eliminate pseudoextreme points, thereby suppressing the oversegmentation phenomenon. The general idea is finding the mark points with the target or image background before the segmentation step. The mark points are regarded as the minimum point of the topographic map to modify thereof whilst shielding other local extreme points. Thus, selecting meaningful mark points becomes the top priority.

The distance transformation image embodies object shape. The binarised image of the cells is transformed into a distance map because the cell is nearly circular, and the number of cells and foreground mark can be obtained by an infinite corrosion operation and area threshold determination. 

*(1) Building Distance Topographic Maps and Extracting Mark Points*. Distance transformation is an operation that converts a binary image into a grey scale image. In this grey scale image, the distance between each pixel and the closest background is represented by the grey level of the pixel. Distance transformation algorithms comprise two main types, namely, non-Euclidean and Euclidean distances [12]. The non-Euclidean distance calculation is simple but is difficult to meet the accuracy requirements. Therefore, Euclidean distance transformation algorithm is used in many applications. The distance map is calculated by using the following formula:

(2) $$\begin{array}{c} I_{Dist}\left(\;i,j\;\right)=255-d_{ij}. \end{array}$$

**Figure 6:**
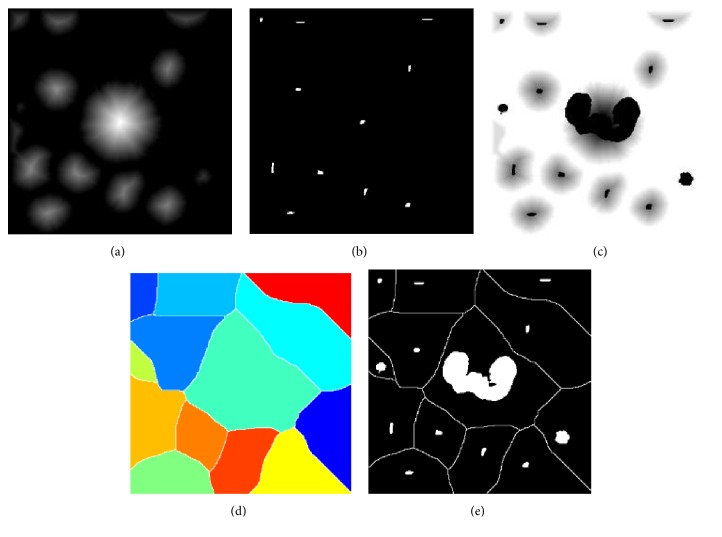
Flowchart of image mark generation: (a) distance topographic maps of cells, (b) foreground marker, (c) modified distance topographic map, (d) colour map of the ridge line after the watershed [the ridge line as the background mark], and (e) *I*_Mark_ [a new marker map is combined with foreground and background markers].

∇*B* is denoised by Gaussian filtering to acquire the distance topographic map *I*_Dist_, where (*i*, *j*) is the Euclidean distance of *d*_*ij*_ coordinate point in the graph, as presented in Figure 6(a). *I*_Dist_ reflects the shape characteristics of the cells. The largest point or point set in each connected region is the central region of individual cells, thereby satisfying the conditions of the foreground marker. The use of distance map to extract the local maximum value thereof can effectively obtain multiple adhesion targets that correspond to numerous foreground markers and thus can effectively separate the adhesion target. The previously obtained WBC markers are also considered foreground markers and are superimposed on the marker map to avoid the impact of the nucleus region on the segmentation algorithm.

*(2) Removing Pseudomarker Points*. In the image of cell adhesion, if most of the overlapping regions exist among the cells, then the overlap region will have multiple local maxima in the distance map. The additional extreme points, that is, pseudomarker points, are not required.

We can use the predetermined cell size information and shape similar to circular geometric information to remove the pseudomarker points. The maximum value of grey scale *d*_max_ in the distance transformation map is obtained. *d*_max_ is the radius of the largest cell in the map and can be used to estimate the cell size. The minimum Euclidean distance between the *n*th mark and its adjacent mark points is denoted by *T*_*n*_. If *T*_*n*_ is smaller than the threshold *T* (e.g., *T* = 2 × *d*_max_), then the *n*th marker point can be judged as a pseudomarker and filtered out, where *n* is smaller than the marker point number.

#### 3.2.3. Background Mark Extraction

The proposed method performs the watershed transformation based on *I*_Dist_ in Figure 6(c) and marks the transformed watershed ridge as the background mark. The result is displayed in Figure 6(d). The watershed ridge is distributed throughout the background area, thereby dividing the image into multiple regions and the adherent cells to form a cell corresponding to a connected region. The overlaid foreground and background markers form a new marker map *I*_Mark_ (Figure 6(e)).

#### 3.2.4. Gradient Topographic Map Reconstruction

The H-minima technique [13] in mathematical morphology is used to modify ∇*I*, thereby suppressing the oversegmentation of watershed transformation. The basic principle is comparing the basin depth of the topographic map with a given threshold *t*_*i*_ to eliminate the local minimum of the basin depth below *t*_*i*_. The process of elimination is similar to the process of filling a shallow basin. The calculation of H-minima is expressed by formulas (3) and (4).

The basin depth *h* is defined as the difference between the minimum gradient values (∇*I*)_min_^Exterier^ and (∇*I*)_min_^Interier^ within the region at the boundary of an area in the image.

(3) $$\begin{array}{c} h={\left(\;\nabla I\;\right)}_{min}^{Exterier}-{\left(\;\nabla I\;\right)}_{min}^{Interier}. \end{array}$$

**Figure 7:**
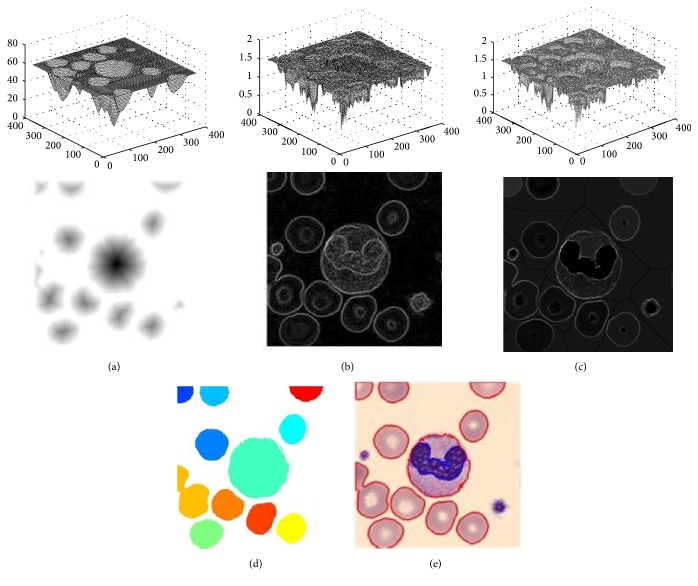
Topographic maps and cell segmentation results: (a) distance topographic maps [2D and 3D], (b) gradient topographic map [2D and 3D], (c) reconstruction map of gradient topographic [2D and 3D], (d) segmented blood cell, and (e) segmented WBCs and RBCs after classification.

H-minima refer to the local minimum of all local minima by suppressing ∇*I*(*i*, *j*). The minimum depth *h* is less than the given *t*_*i*_ and can be achieved by using the following formulas:

(4) ∇T=R∇Ierode∇I+ti−∇I,∇Imark=0∇Tp≤0,  p∈∇I1∇Tp>0,  p∈∇I,

where *R*_∇*I*_^erode^(∇*I* + *t*_*i*_) indicates that ∇*I*(*i*, *j*) is corroded according to ∇*I* + *t*_*i*_ of the image. The range of *t*_*i*_ is generally between 2 and 4. Therefore, we select 3. The local minimum in the corrected gradient topographic image will only exist at the marker location. A foreground marker corresponds to a cell, and the background marker is distributed on the image background, thus forming a one-to-one relationship which can suppress the oversegmentation phenomenon. The modified gradient image ∇*I*_mark_ is presented in Figure 7(c).

Figure 7 depicts the 2D and 3D representations of the three types of topographic maps, where (a) graph is the distance topographic map *I*_Dist_, (b) graph is the unmodified gradient topographic map ∇*I*, and (c) graph is the modified gradient topographic map ∇*I*_mark_. Compared with map (b), map (c) exhibits the edge of the formation of the cell peak top and the cell interior and background areas for the trough terrain.

The marker-controlled watershed algorithm based on ∇*I*_mark_ is performed to obtain the segmentation result, such as the colour map illustrated in Figure 7(e), given the blood cell segmentation.

### 3.3. Classification

In Section 3.2, the segmented blood cell is obtained through foreground, background mark extraction, gradient topographic map reconstruction, and marker-controlled watershed. In order to further distinguish which blood cells are WBCs or RBCs, it can be based on the characteristics of WBCs that have nuclei and RBCs without nuclei. We classify the cells according to the WBC markers obtained in Section 3.1.

As shown in Figure 7(e), the cells that contain blue lines that represent the cell nuclei are WBCs and the other cells are RBCs.

## 4. Experimental Results

Numerous microscopic images with 760 × 567 pixels are used to test and verify the timeliness and robustness of the proposed method. All test images are downloaded from the CellAtlas application. A dataset consists of 100 test images. The experimental results are summarized in Tables 1 and 2 to prove the performance of the proposed method. The proposed method obtains the contours of WBCs and RBCs simultaneously, whereas other methods can only acquire the contours of either WBCs or RBCs. The statistical indicators of WBCs and RBCs are thus calculated. The main purpose of Experiment 1 is testing the time complexity of the method, and the aim of Experiment 2 is verifying the robustness of the method. In Experiment 1, the average consumption time of the method for 100 cells is counted to compare the time complexity of multiple methods. The proposed method can obtain two types of cells; hence, the statistical data of 50 of the two types of cells are counted. The results are as follows.

Table 1 implies that the proposed method is the least average time-consuming method compared with other methods under the same conditions. The proposed method does not achieve a significant time advantage because this method uses two watershed operations. However, the method can also be effective for two cell types simultaneously, and two algorithms are not required to handle two cell types. Therefore, the image processing is simplified.

In Experiment 2, the automatically segmented results are compared with the results obtained manually by a haematologist. The proposed method is consistent and coherent in datasets, with the accuracy rates of the WBC segmentation at 97.2% and RBCs at 94.8%. In this evaluation, the segmentation result is considered accurate when the automatically detected boundary closely matches the manually traced boundary.

The proposed method has a greater advantage than the existing algorithms in statistical performance, especially in the undersegmentation index. In the statistical data of our methods, the one-to-one rate of WBCs is 97.2%, and the RBC segmentation accuracy rate is 94.8%. The oversegmentation and undersegmentation rates of RBCs are 3% and 1%, correspondingly. The results of extracting WBCs and RBCs separately are compared with the above algorithm results. We also obtain a small advantage. Our “fault” rate is the lowest, thereby proving the algorithm's robustness.

Table 2 indicates that the proposed method is tested on the database with satisfactory results, especially the undersegmentation. The oversegmentation and undersegmentation rates of RBCs are 3% and 1%, respectively. The “fault” rate of our method is the lowest. These statistical data show that the proposed method is robust.

**Figure 8:**
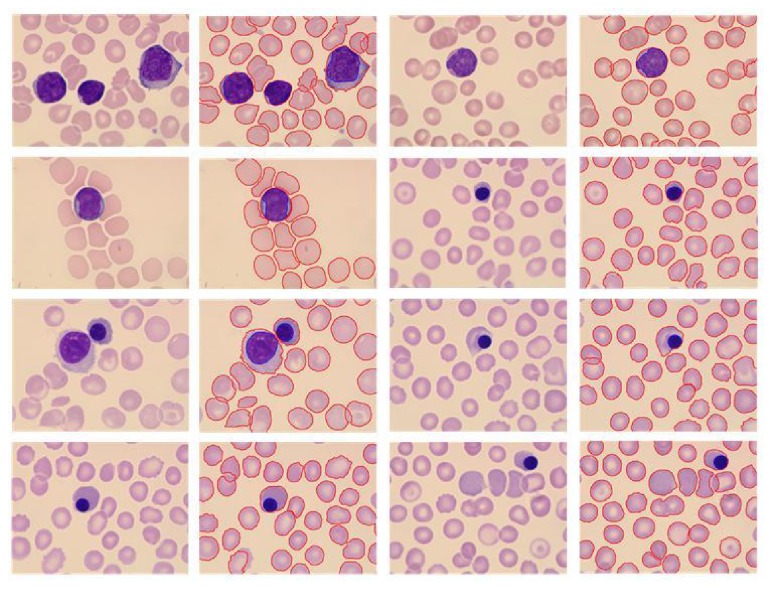
Example of the proposed algorithm's segmentation results.

The segmentation results are presented in Figure 8, where the red line is the outline of the blood cells, the blue line is the nucleus contours, and the cells containing the nucleus are WBCs. The test images comprise an image containing a plurality of WBCs. For these special cases, the method can effectively obtain the cytoplasm and nucleus. The method also obtains satisfactory segmentation results for RBC images.

## 5. Conclusions

In this article, we present a new method for simultaneous segmentation of WBCs and RBCs in blood smear images. We model the edge gradient and shape characteristics of these cells by defining two transformations and efficiently using these transformations in a marker-controlled watershed. The experiments show that this method can simultaneously extract the two blood cell types to simplify operations and reduce computing time. However, the proposed method must be further improved because producing slices and other causes of cell adhesion is serious, and the moderate image quality can negatively impact the accuracy of the segmentation algorithm.

## Figures and Tables

**Table 1:** Algorithm time complexity.

Method	Time (s)
Cuevas et al.'s method [5]	0.30
Wei and Cao's method [9]	0.46
Proposed method	0.32

**Table 2:** Segmentation performance.

Method	Cell	One-to-one	Overseg.	Underseg.	Fault
Cuevas et al.'s method [5]	WBCs	96.2%	0.8%	2.4%	1.0%
Wei and Cao's method [9]	RBCs	94.0%	2.0%	3.0%	1.0%
Proposed method	WBCs	97.2%	1.0%	1.8%	0.0%
	RBCs	94.8%	3.0%	1.0%	0.8%
